# Functions and Therapeutic Potentials of Long Noncoding RNA in Skeletal Muscle Atrophy and Dystrophy

**DOI:** 10.1002/jcsm.13747

**Published:** 2025-03-04

**Authors:** Yidi Zhang, Teng Wang, Ziang Wang, Xin'e Shi, Jianjun Jin

**Affiliations:** ^1^ Laboratory of Animal Fat Deposition and Muscle Development, Key Laboratory of Animal Genetics, Breeding and Reproduction of Shaanxi Province, College of Animal Science and Technology Northwest A&F University Yangling China

**Keywords:** long noncoding RNAs, muscle atrophy, muscular dystrophy, protein synthesis and degradation, therapeutic implications

## Abstract

Skeletal muscle is the most abundant tissue in the human body and is responsible for movement, metabolism, energy production and longevity. Muscle atrophy is a frequent complication of several diseases and occurs when protein degradation exceeds protein synthesis. Genetics, ageing, nerve injury, weightlessness, cancer, chronic diseases, the accumulation of metabolic byproducts and other stimuli can lead to muscle atrophy. Muscular dystrophy is a neuromuscular disorder, part of which is caused by the deficiency of dystrophin protein and is mostly related to genetics. Muscle atrophy and muscular dystrophy are accompanied by dynamic changes in transcriptomic, translational and epigenetic regulation. Multiple signalling pathways, such as the transforming growth factor‐β (TGF‐β) signalling pathway, the phosphatidylinositol 3‐kinase (PI3K)/protein kinase B (AKT)/mechanistic target of rapamycin (mTOR) pathway, inflammatory signalling pathways, neuromechanical signalling pathways, endoplasmic reticulum stress and glucocorticoids signalling pathways, regulate muscle atrophy. A large number of long noncoding RNAs (lncRNAs) have been found to be abnormally expressed in atrophic muscles and dystrophic muscles and regulate the balance of muscle protein synthesis and degradation or dystrophin protein expression. These lncRNAs may serve as potential targets for treating muscle atrophy and muscular dystrophy. In this review, we summarized the known lncRNAs related to muscular dystrophy and muscle atrophy induced by denervation, ageing, weightlessness, cachexia and abnormal myogenesis, along with their molecular mechanisms. Finally, we explored the potential of using these lncRNAs as therapeutic targets for muscle atrophy and muscular dystrophy, including the methods of discovery and clinical application prospects for functional lncRNAs.

## 1 Introduction

Skeletal muscle is widely distributed in the human body, accounting for about 40% of total body mass [1]. The presence of skeletal muscle enables individuals to move freely, expand the range of motion and improve survival. In addition, skeletal muscle is also necessary to maintain the basic biological processes of the human body, especially glucose oxidation, gluconeogenesis and glycogen storage, correlating with energy supply. Skeletal muscle originates from paraxial mesoderm and develops from myogenic progenitor cells (MPCs) that can be differentiated from the cells residing in dermomyotome [2]. Subsequently, MPCs migrate to extremities and further differentiate and mutually fuse to form myofibres. These processes are attributed to myogenic regulatory factors [3], Sonic Hedgehog pathway [4], Wnt pathway [5] and transcription factors like paired box 3 (PAX3) and PAX7 [6]. Postnatal skeletal muscle growth depends primarily on muscle protein deposition. However, skeletal muscle growth sometimes goes in the opposite direction of muscle hypertrophy because of additional stimuli that reduce dystrophin protein expression or disrupt the balance between muscle protein synthesis and degradation and thus leads to muscular dystrophy and muscle atrophy. For instance, patients with obesity‐related type 2 diabetes have been proven to have muscle atrophy because of the presence of inflammatory responses [7] and metabolic disorders [8]. Most cancer types are also accompanied by loss of muscle mass, impaired mobility and reduced quality of life. Under normal physiological conditions, ageing is a potential trigger for skeletal muscle atrophy. It was reported that the global prevalence of sarcopenia ranges from 8% to 36% in individuals under the age of 60 and from 10% to 27% in individuals aged 60 and above [9]. Sarcopenia can easily lead to falls in elderly people. With various pathogenic factors and widespread prevalence, muscle atrophy and muscular dystrophy are becoming a great challenge for clinical treatment and an economic burden on healthcare systems.

Long noncoding RNAs (lncRNAs) are RNA transcripts with a length of more than 200 nucleotides, and they are highly cell specific and less conserved compared with protein‐coding genes [10]. Although lncRNAs, as RNA molecules, usually cannot encode proteins, they have unique biological functions and diverse molecular mechanisms. LncRNAs are emerging as a new layer of regulation for various biological processes. Our results and those of other researchers have proved that lncRNAs play critical roles in embryonic or postnatal muscle development [11], muscle regeneration [12] and muscle wasting progression. They modulate skeletal muscle mass and functions by activating muscle satellite cells, regulating the proliferation and differentiation of myoblasts, transforming muscle fibre types, maintaining muscle protein balance and regulating the expression of dystrophin protein. This article mainly summarizes the functions and molecular mechanisms of known lncRNAs in muscular dystrophy and muscle atrophy induced by various factors, including denervation, ageing, weightlessness, cachexia and abnormal myogenesis. The possibility of targeting lncRNAs was also discussed for the treatment of muscular atrophy and dystrophy.

## 2 Molecular Mechanisms and Functions of LncRNA

According to the position on the genome, lncRNAs can be divided into intergenic lncRNAs, antisense lncRNAs, sense lncRNAs, intronic lncRNAs, bi‐directional lncRNAs and enhancer lncRNAs [13]. The complexity of lncRNA function lies in its ability to go beyond transcriptional regulation of the genome and exert effects at the epigenetic modification, post‐transcriptional and chromatin levels. The molecular mechanisms of lncRNAs can be categorized into four types [14]: (A) lncRNAs can act as signal molecules that transmit external stimulus signals or serve as markers of space, time and transcript abundance to regulate important biological processes in cells. (B) lncRNAs can act as guide molecules that direct ribonucleoprotein complexes to specific targets, thereby influencing gene expression. (C) lncRNAs can act as scaffold molecules that facilitate the assembly of RNA‐protein complexes. (D) lncRNAs can act as decoy molecules that competitively bind transcription factors, chromatin modifiers, miRNAs or other regulatory factors. The subcellular localization of lncRNAs also determines their functions. A recent study demonstrated that lncRNAs present in the nucleus can serve as a part of chromatin, recruiting chromatin modifying proteins to help form active or inactive regions of chromatin [15]. Some lncRNAs can influence chromatin architecture by participating in chromatin dynamics formation. A study found that lncRNA *ThymoD* could promote demethylation of the CTCF (DNA‐binding protein CCCTC‐binding factor) binding site in T cell chromatin, activating cohesin‐dependent looping that repositions the Bcl11b (Bcl11 transcription factor b) enhancer from the nuclear lamina to the interior. This repositioning results in the enhancer being closer to the promoter, preventing the development of T cells into malignant lymphoma [16]. LncRNAs in the cytoplasm have a wider range of functions. They can affect the polymerization and depolymerization processes of the cytoskeleton, for example, *LNC CRYBG3* can bind to G‐actin to inhibit its polymerization and contraction ring formation, leading to M‐phase cell arrest [17]. LncRNA can also participate in mRNA translation and protein modification. Recent research found that lncRNAs can exert more functions by translating peptides [18]. Meanwhile, lncRNAs also participate in mRNA alternative splicing, and their molecular mechanism of regulating mRNA transcription is well known [19]. In addition, the functions of lncRNAs localized in mitochondria, endoplasmic reticulum, Golgi apparatus and plasma membrane should not be underestimated. For example, *LINC00473* can crosslink with mitochondria and lipid droplet proteins, thereby influencing lipolysis and mitochondrial oxidative metabolism [20]. Over the past decades, from being regarded as ‘transcriptional noise’ to becoming indispensable regulators, lncRNAs have emerged as a crucial regulatory layer in various biological processes because of their diverse functions and mechanisms. In recent years, research on lncRNAs has highlighted their potential as therapeutic targets, showing that lncRNAs hold significant promise for future disease treatments.

## 3 Molecular Mechanisms Regulating Muscle Atrophy

### 3.1 Signalling Pathway Mediating Protein Degradation in Skeletal Muscle

**FIGURE 1:**
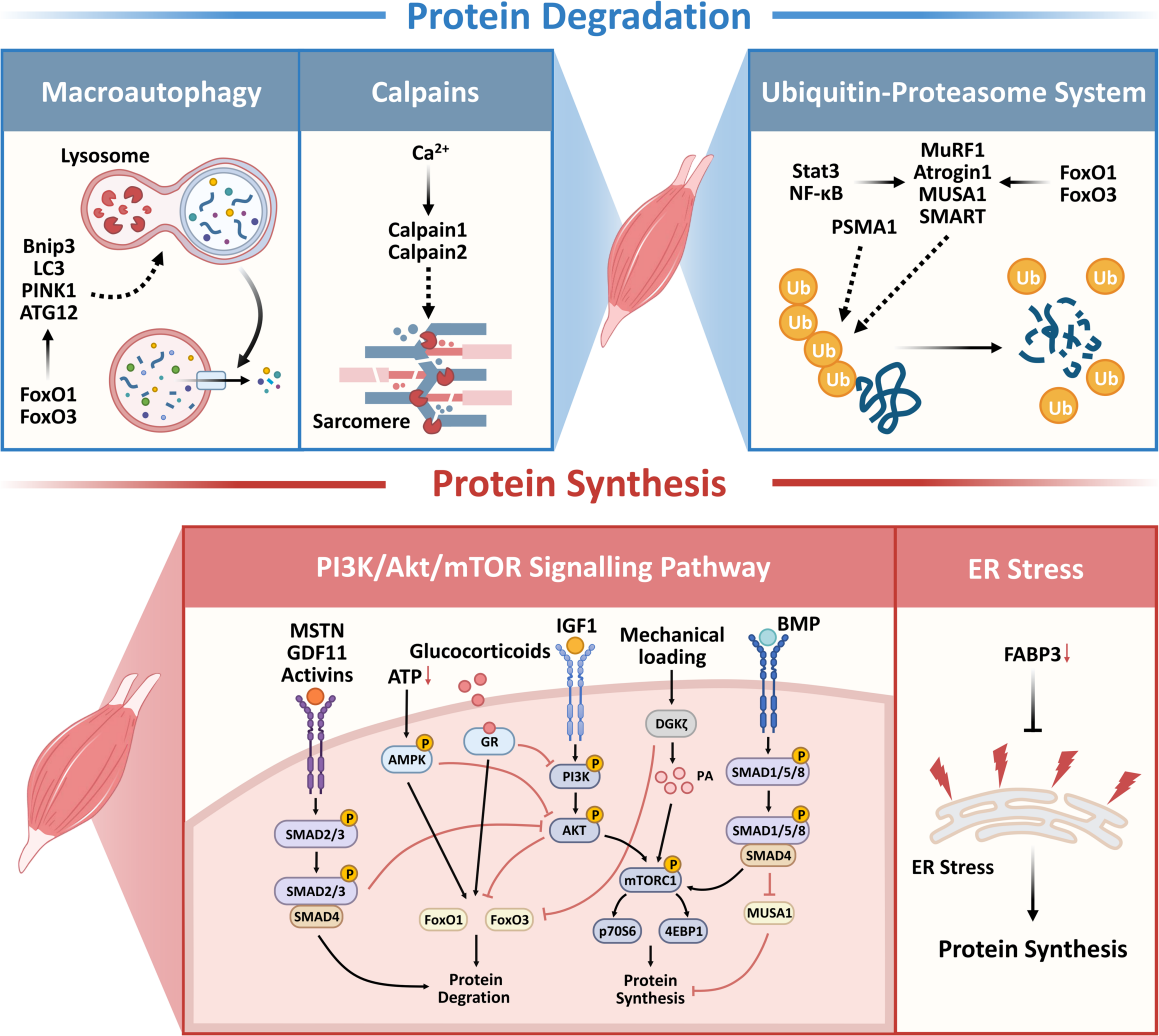
The mechanisms of protein degradation and synthesis in muscles. Proteins in muscles are degraded through three pathways: macroautophagy, calpains and proteasome ubiquitination. The protein synthesis pathway in muscles involves the endoplasmic reticulum stress and the PI3K/AKT/mTOR signalling pathway. PI3K/AKT/mTOR signalling pathway is modulated by various factors, including MSTN, GDF11, activins, ATP levels, glucocorticoids, IGF‐1, mechanical loading and BMP. The dotted line indicates participation in the process. MSTN, myostatin. GDF11, growth differentiation factor‐11. IGF‐1, insulin‐like growth factor 1. BMP, bone morphogenetic protein. GR, glucocorticoids receptor. DGKζ, diacylglycerol kinase ζ. PA, phosphatidic acid. ATP, adenosine‐5′‐triphosphate.

Proteins in skeletal muscle are degraded through three main pathways: the ubiquitin‐proteasome system (UPS), the autophagy‐lysosomal pathway (ALP) and calpains (Figure 1). The components that participate in the UPS process include ubiquitin‐activating enzyme, ubiquitin‐conjugating enzyme, ubiquitin‐protein ligase, ubiquitin and the 26S proteasome [21]. They are jointly involved in the ubiquitination degradation process. Ubiquitin‐E3 ligase muscle ring finger‐1 (MuRF1) and muscle atrophy F‐box (Atrogin‐1) are the two ligases that exist only in muscle and play a major role in most types of muscle atrophy [22]. Muscle ubiquitin ligase of the SCF complex in atrophy‐1 (MUSA1) and specific of muscle atrophy and regulated by transcription (SMART) play a critical role in protein ubiquitination following denervation [23, 24]. PSMA1 (proteasome subunit alpha type 1) contributes to immobilization‐induced muscle atrophy [25]. The ALP is an evolutionarily conserved catabolic process responsible for maintaining intracellular homeostasis. It functions by encapsulating damaged organelles, protein aggregates or lipid droplets within autophagosomes, which subsequently fuse with lysosomes to degrade these contents [26]. There are three different ways to deliver substrates to lysosomes: macroautophagy, microautophagy and chaperone‐mediated autophagy [27]. However, only macroautophagy plays a major role in skeletal muscle [28]. BNIP3 (Bcl‐2/adenovirus E1B 19‐kDa interacting protein), PTEN‐induced kinase 1 (PINK1), LC3 (light chain 3), GABARAP (GABA type A receptor–associated protein) and ATG12 (autophagy related 12) are the key proteins involved in the autophagy pathway [29]. Notably, they are co‐regulated with MuRF1, Atrogin‐1, MUSA1 and SMART by transcription factors forkhead box O1 (FoxO1) and FoxO3, which are members of the forkhead box family [30, 31]. Calpains are intracellular Ca^2+^‐regulated cysteine proteases. There are three types of calpains involved in muscle atrophy: ubiquitous calpains 1 and 2 and calpain 3. Ubiquitous calpains 1 and 2 destroy the Z‐disk, releasing actin and myosin to promote their better degradation, whereas the deficiency of calpain 3 induces muscle atrophy [32].

Several signalling pathways can mediate protein degradation (Figure 1). Myostatin, growth differentiation factor‐11 (GDF11) and activin are members of the TGF‐β superfamily [22]. They promote the expression levels of genes related to the ubiquitination pathway by inducing phosphorylation of the SMAD family member 2 and 3 (SMAD2/3) complex that inhibits the AKT–mTOR pathway [22]. Glucocorticoids bind to intracellular receptors and increase downstream gene expression, including the expression of *FoxO1* and *FoxO3*, to inhibit the AKT–mTOR pathway [33]. Surely, there are numerous AKT‐independent regulation pathways. For instance, the AMPK (AMP‐activated protein kinase) pathway depends on ATP (adenosine‐5′‐triphosphate) level can directly promote FoxO3 nuclear translocation and enhance its transcriptional activities [34]. Nuclear factor kappa‐B (NF‐κB) can directly enhance the transcription of *MuRF1* [35], mediating the effect of inflammatory cytokines, such as TNF‐α (tumour necrosis factor‐α), interleukin‐6 (IL‐6) and TWEAK (tumour necrosis factor‐like weak inducer of apoptosis), on muscle atrophy [22]. IL‐6 activated p‐Stat3 can stimulate the expression of *C/EBPδ* (CCAAT/enhancer‐binding protein delta), leading to the upregulation of Atrogin‐1, MuRF‐1 and myostatin [36]. During muscle atrophy induced by starvation, immobilization or ageing, the expression of activating transcription factor 4 (ATF4) increases [37]. ATF4 binds to *C/EBPβ* to promote the expression of growth arrest and DNA damage inducible α (*Gadd45α*) [38]. *HDAC4* expression increases after denervation and promotes muscle atrophy by promoting myogenin and *Gadd45α* expression [37]. Gadd45α is essential for muscle atrophy especially in acute stress conditions, and FoxO1 and FoxO3 can promote its transcription [39]. However, the downstream mechanism by which Gadd45α promotes muscle atrophy remains unclear. In addition, mitochondrial energy metabolism disorders are central to the pathogenesis of muscular dystrophy, disuse atrophy and sarcopenia [40].

### 3.2 Signalling Pathway Mediating Protein Synthesis in Skeletal Muscle

The phosphatidylinositol 3‐kinase (PI3K)/protein kinase B (AKT)/mechanistic target of rapamycin (mTOR) pathway is the most representative one to regulate protein synthesis (Figure 1). AKT phosphorylates FoxO3 to inhibit its translocation from the cytoplasm to the nucleus and further inhibit the upregulation of MuRF1 and Atrogin‐1. Moreover, AKT promotes protein synthesis by phosphorylating mechanistic target of rapamycin complex 1 (mTORC1). mTORC1 is a serine/threonine protein kinase in the PI3K‐related kinase family [41] and is essential for muscle protein synthesis. p70S6K (70‐kDa ribosomal protein S6 kinase) and translation initiation factor 4E binding protein 1 (4EBP‐1) are key downstream targets of mTORC1. The mTORC1 phosphorylates the hydrophobic motif site Thr389 of ribosomal protein S6 kinase beta 1 (S6K1) [42]. Subsequently, S6K1 is phosphorylated and activated by PDK1 (3‐phosphoinositide‐dependent protein kinase 1), thereby phosphorylating and activating several substrates to promote translation initiation [42]. The mTORC1 also phosphorylates multiple sites of 4EBP‐1 to promote the dissociation from translation initiation factor 4E (eIF4E) and thus promote translation by abolishing the suppressive effect of the eIF4F complex [43]. Furthermore, mTORC1 inhibits the critical proteins ULK1 (unc‐51 like autophagy activating kinase 1) in the ALP [44]. Insulin like growth factor 1 (IGF‐1) can induce muscle hypertrophy by binding to IGF‐1 receptor to activate the PI3K/AKT/mTOR pathway [45]. Bone morphogenetic protein (BMP) is a member of the TGF‐β family. It induces the formation of the SMAD1/4/5/8 complex to activate mTORC1 and promote protein synthesis [22]. Endoplasmic reticulum (ER) stress blocks protein translation through the PERK (STING‐PKR‐like endoplasmic reticulum kinase)/eIF2α (eukaryotic initiation factor 2 alpha) pathway to cause muscle atrophy during ageing [46] (Figure 1). Reducing ER stress improves protein synthesis [46]. During mechanical loading, DGKζ (diacylglycerol kinase ζ) suppresses FoxO3 activity and promotes the synthesis of PA (phosphatidic acid), which is an mTOR agonist, to promote protein synthesis [47].

## 4 The Function and Mechanism of LncRNA in Muscle Atrophy and Dystrophy

**FIGURE 2:**
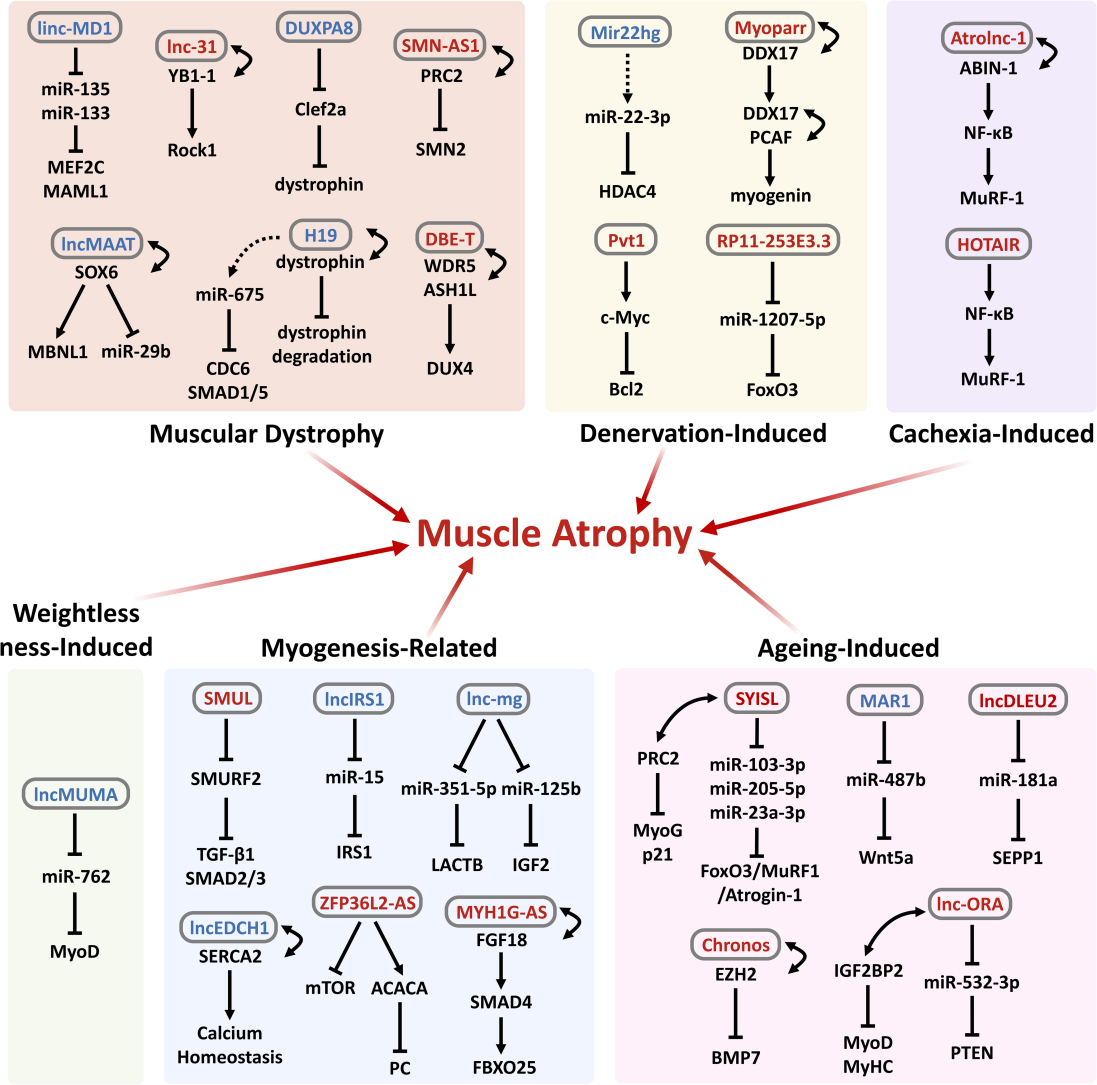
The mechanisms of lncRNAs involved in regulating muscle atrophy. The blue font indicates inhibition of muscle atrophy, the red font indicates promotion of muscle atrophy, the dashed arrow represents the production of miRNA by lncRNA, and the bidirectional arrow represents the interaction between lncRNA and proteins.

To date, thousands of differentially expressed lncRNAs have been identified in muscle spanning from healthy muscle to dystrophy or muscle atrophy. In our previous study, we identified 1913 differentially expressed lncRNAs during myoblast differentiation and identified a key lncRNA named *SYISL* that can promote dexamethasone (Dex) induced muscle atrophy and sarcopenia [48]. In addition, other studies have also found many lncRNAs related to muscle atrophy. These above results indicated that a large amount of lncRNAs are abnormally expressed during muscle atrophy and participate in the process of muscle atrophy caused by different inducing factors (Figure 2) (Table 1).

**TABLE 1:** Examples of lncRNAs that have functions in muscle atrophy.

LncRNA	Type of muscle atrophy	Changes in level	Function location	Regulation method	Reference
*linc‐MD1*	Muscular dystrophy	Downregulated in the muscles of DMD mouse models.	Cytoplasm	Adsorption of *miR‐135* and *miR‐133*.	[49, 50]
*lnc‐31*	Muscular dystrophy	Upregulated in proliferating myoblasts.	Cytoplasm	Bind to YB‐1 protein to enhance its stability.	[51]
*DUXAP8*	Muscular dystrophy	Downregulated in the muscles of DMD patients.	Nucleus	Promote epigenetic regulation of *Celf2a* coding region.	[52]
*H19*	Muscular dystrophy	Downregulated in the muscles after birth.	Cytoplasm	Generate miR‐675‐3p and miR‐675‐5p; competitive binding of muscular dystrophy protein to prevent its degradation.	[53, 54]
*DBE‐T*	Muscular dystrophy	Upregulated in the muscles of FSHD patients.	Nucleus	Recruit ASH1L protein and WDR5 protein to FSHD locus.	[55, 56, 57]
*SMN‐AS1*	Muscular dystrophy	Upregulated in the neurons of SMA patients.	Nucleus	Promote the recruitment of the PRC2 to the *SMN2* promoter.	[58]
*lncMAAT*	Muscular dystrophy	Downregulated in various muscle atrophy.	Nucleus	Promote the expression of MBNL1 in a cis manner; Binding to SOX6 to inhibit its transcriptional activity.	[59, 60]
*Mir22hg*	Denervation‐induced muscle atrophy	Upregulated during the progression of myogenesis.	Cytoplasm	Generate *miR‐22‐3p*.	[61]
*Myoparr*	Denervation‐induced muscle atrophy	Co‐expressed with myogenin.	Nucleus	Enhance DDX17‐PCAF interaction.	[62]
*Pvt1*	Denervation‐induced muscle atrophy	Upregulated in the early stage of both models.	Cytoplasm	Increase the stability of c‐Myc.	[63]
*RP11‐253E3.3*	Denervation‐induced muscle atrophy	Upregulated in the muscles of SCI patients.	Cytoplasm	Adsorption of *miR‐1207‐5p*.	[64]
*PRKG1‐AS1*	Ageing‐induced muscle atrophy	Upregulated in elderly muscles.	\	\	[65]
*lncDLEU2*	Ageing‐induced muscle atrophy	Upregulated in the muscles of sarcopenia models.	Cytoplasm	Adsorption of *miR‐181a*.	[66]
*SYISL*	Ageing‐induced muscle atrophy	Upregulated in various muscle atrophy.	Cytoplasm; Nucleus	Adsorption of *miR‐103‐3p*, *miR‐205‐5p* and *miR‐23a‐3p*; promote the recruitment of the PRC2 to the *p21* and myogenin gene promoter.	[48, 67]
*Chronos*	Ageing‐induced muscle atrophy	Upregulated in the muscles with ageing.	Nucleus	Recruit EZH2 to the *BMP7* locus.	[68]
*MAR1*	Ageing‐induced muscle atrophy	Downregulated in the muscles of elderly mice.	Cytoplasm	Adsorption of *miR‐487b*.	[69]
*lnc‐ORA*	Ageing‐induced muscle atrophy	Upregulated in the muscles of elderly mice.	Cytoplasm	Adsorption of *miR‐532‐3p*; Competitively binding IGF2BP2 to reduce the stability of myogenesis genes.	[70, 71]
*lncMUMA*	Weightlessness‐induced muscle atrophy	Downregulated in the muscles of HLS mice.	Cytoplasm	Adsorption of *miR‐762*.	[72]
*Atrolnc‐1*	Cachexia‐induced muscle atrophy	Upregulated in the muscles of CKD patients.	Cytoplasm	Bind to NF‐κB inhibitory factor ABIN‐1.	[73]
*HOTAIR*	Cachexia‐induced muscle atrophy	Upregulated in bladder tumours following cisplatin treatment.	Cytoplasm	Promote NF‐κB inhibitory factor IκBα degradation.	[74]
*LncEDCH1*	Myogenesis	Downregulated during myogenic differentiation.	Cytoplasm	Bind to SERCA2 protein to enhance its activity and stability.	[75]
*ZFP36L2‐AS*	Myogenesis	Upregulated in the muscles of fast‐growth‐rate broiler chickens.	Cytoplasm	Interact with ACACA and PC to enhance ACACA activity and facilitate PC degradation.	[76]
*LncIRS1*	Myogenesis	Upregulated in the muscles of fast‐growth‐rate broiler chickens.	Cytoplasm	Adsorption of *miR‐15*.	[77]
*SMUL*	Myogenesis	Downregulated during the process of myoblast differentiation.	Cytoplasm	Disrupt stability of SMURF2 mRNA via nonsense‐mediated mRNA decay.	[78]
*Lnc‐mg*	Myogenesis	Upregulated during the differentiation of MuSCs.	Cytoplasm	Adsorption of *miR‐351‐5p* and *miR‐125b*.	[79, 80]
*MYH1G‐AS*	Myogenesis	Upregulated in fast‐twitch fibres.	Nucleus	Inhibit FGF18 protein stabilization to reduce the interaction of FGF18 to SMARCA5	[11]

*Note:* YB‐1, Y‐box binding protein 1. Celf2a, CUGBP Elav‐like family member 2a. ASH1L, absent, small, or homeotic1‐like. WDR5, WD repeat domain 5. FSHD, facioscapulohumeral muscular dystrophy. PRC2, polycomb repressive complex 2. SMN2, survival motor neuron 2. MBNL1, muscleblind‐like 1. SOX6, SRY‐box transcription factor 6. DDX17, DEAD‐box RNA helicase 17. PCAF, p300/CBP‐associated factor. c‐Myc, Myc proto‐oncogene protein. EZH2, enhancer of zeste homologue 2. BMP7, bone morphogenetic protein 7. IGF2BP2, insulin‐like growth factor 2 mRNA‐binding protein 2. NF‐κB, nuclear factor kappa‐B. SERCA2, sarcoendoplasmic reticulum Ca2+ ATPase‐2. ACACA, acetyl‐CoA carboxylase alpha. PC, pyruvate carboxylase. SMURF2, SMAD‐specific E3 ubiquitin ligase 2. FGF18, fibroblast growth factor 18. SMARCA5, SWI/SNF related, matrix associated, actin dependent regulator of chromatin, subfamily a, member 5.

### 4.1 LncRNA in Muscular Dystrophy

Duchenne muscular dystrophy (DMD) is an X‐linked recessive neuromuscular disease caused by mutations of genes that encode dystrophin protein. Xu et al. identified several differentially expressed lncRNAs in healthy individuals and patients with DMD, including *AL132709*, *LINC00310*, *ALDH1L1‐AS2* and *XIST* [81]. Bovolenta et al. reported several lncRNAs, such as *ncNT44s* and *ncNT55s*, which were specifically transcribed from the DMD locus and are able to promote the orchestration and homeostasis of the muscle dystrophin expression pattern by targeting the dystrophin promoter transcriptional activity [82]. These lncRNAs may be potential targets for DMD treatment.

*linc‐MD1* is the first muscle‐specifically expressed lncRNA found to be associated with DMD and has low expression levels in DMD mouse models [49]. *linc‐MD1* can adsorb *miR‐135* and *miR‐133* by acting as a molecular sponge, thereby relieving their targeted inhibition of *MAML1* (mastermind‐like 1) and myocyte enhancer factor 2C (*MEF2C*) genes and promoting myogenesis [49, 50]. *miR‐31* exists in DMD patients at a high level, and it inhibits the expression of dystrophin [83]. *lnc‐31* and *miR‐31* are transcribed from the same nuclear precursor and are produced by different splicing patterns, both are highly expressed in proliferating myoblasts and strongly downregulated during differentiation [84]. This finding indicated that *lnc‐31* may be involved in the process of the expression of muscular dystrophy proteins inhibited by *miR‐31*. Furthermore, *lnc‐31* is associated with delayed differentiation of myoblasts in DMD [84]. Rho‐associated coiled‐coil‐containing protein kinases 1 (Rock1) is a known protein that inhibits myogenic differentiation [51, 85]. Dimartino et al. found that *lnc‐31* promotes the translation of *Rock1* by binding to YB‐1 (Y‐box binding protein 1) protein [51].

Exon skipping is one of the most prominent approaches for the treatment of DMD. The decrease of lncRNA *DUXAP8* in DMD patients is accompanied by an increase in CUGBP Elav‐like family member 2a (Celf2a), which leads to an abnormal skipping of dystrophin exon 45 and thus inhibits dystrophin protein synthesis [52]. On the contrary, overexpression of *DUXAP8* can epigenetically inhibit the expression of *Celf2a*, promote the skipping of dystrophin exon 45 and restore the expression of the dystrophin gene [52].

*H19* is a widely known genomically imprinted lncRNA with high expression levels in the embryonic stage but downregulated after birth [86]. *H19* acts as a molecular sponge of *miR‐675‐3p* and *miR‐675‐5p*, which down‐regulate *SMAD1*, *SMAD5* and *CDC6* (cell division cycle 6), to promote myogenesis [53]. *H19* shows therapeutic potential for DMD. The AT‐rich motifs of *H19* can bind to the Zinc finger domain of dystrophin to repress its degradation by competing with MuRF1 protein. Cell experiments showed that the mutations in the AT‐rich motifs of *H19* resulted in a decrease in the level of dystrophin [54].

Facioscapulohumeral muscular dystrophy (FSHD) is an autosomal dominant disorder [87]. The pathogenesis of FSHD is that a reduced copy number of D4Z4 located at chromatin 4q35 results in reduced methylation and inhibitory heterochromatin levels in this region, which in turn promotes the abnormal expression of sequence‐specific transcription factor double homeobox 4 (DUX4) protein, a key factor to induce muscle atrophy in FSHD [87]. Cabianca et al. discovered that lncRNA *DBE‐T* is selectively produced in patients with FSHD, and it is associated with the disinhibition of the *DUX4* gene [55]. The histone methyltransferase ASH1L (absent, small or homeotic1‐like), known to specifically methylate histones H3K4 and H3K36, is preferentially recruited by *DBE‐T* to promote the disinhibitory state of the DUX4 gene through remoting control [55, 56]. Additionally, WDR5 (WD repeat domain 5) binds directly to *DBE‐T* and is required for the expression of the *DUX4* gene [57].

Spinal muscular atrophy (SMA) is a neuromuscular disease characterized by muscular weakness or even death because of the insufficient level of survival motor neuron (SMN) protein. SMN protein is usually encoded by the *SMN1* gene. Mutations in the *SMN1* gene in SMA patients cannot produce normal proteins, but *SMN2*, as a highly homologous gene of *SMN1*, can compensate for SMN1. However, *SMN2* often generates dysfunctional proteins because of exon 7 skipping during alternative splicing, which is caused by a single‐nucleotide C‐to‐T conversion [88]. LncRNA *SMN‐AS1* is highly enriched in neurons and originates from a central region of intron 1 of the *SMN* genes [58]. *SMN‐AS1* promotes the recruitment of the polycomb repressive complex 2 (PRC2) to the *SMN2* promoter to suppress *SMN2* expression.

LncRNA *MAAT* is down‐regulated in muscle atrophy induced by a variety of in vivo or in vitro factors [59]. The gene of *lncMAAT* is located in the third intron of *MBNL1* (muscleblind‐like 1) gene. The protein product of *MBNL1* gene have been reported to reverse the proliferation defect of muscle satellite cells in dystrophy type 1 by disturbing RNA‐splicing function [59, 60]. Here, the inhibition of *lncMAAT* represses the expression of MBNL1 by a cis‐regulatory module and thus promotes muscle atrophy [59]. *LncMAAT* can also interact with the transcription factor SOX6 (SRY‐box transcription factor 6) to inhibit the transcription of *miR‐29b*, thereby suppressing muscle atrophy [59].

### 4.2 LncRNA in Denervation‐Induced Muscle Atrophy

The function and metabolic homeostasis of muscles are greatly influenced by peripheral nerves. Generally, the impact of nerve injury on the development of normal muscles towards muscle atrophy is irreversible. Histone deacetylase 4 (HDAC4) inhibition is assumed to alleviate denervation‐induced muscle atrophy [37]. A newly identified lncRNA, *Mir22hg*, associated with the inhibition of *HDAC4* was found. *Mir22hg* can inhibit *HDAC4* by producing *miR‐22‐3p* [61]. In muscle, *Mir22hg* knockdown significantly reduces muscle mass and delays the regeneration and repair processes after injury [61].

LncRNA *Myoparr* is transcribed from the upstream region of the myogenin gene. It has similar expression patterns but divergent promoters [62]. *Myoparr* is enriched only in the nucleus. Interference with *Myoparr* significantly reduces the level of myogenin. Research on the mechanism has proved that *Myoparr* interacts with DDX17 (DEAD‐box RNA helicase 17) to promote the interaction between DDX17 and PCAF (p300/CBP‐associated factor), thereby promoting the expression of myogenin [62]. In addition, the expression of myogenin is repressed by innervation during skeletal muscle maturation, and it can accelerate skeletal muscle atrophy after denervation [89]. Knockdown of *Myoparr* significantly reduces the myogenin level and blocks denervation‐induced skeletal muscle atrophy in mice [62].

Alessio et al. established denervation and amyotrophic lateral sclerosis mouse models, and they found that *Pvt1* increased significantly in the early stage of both two types models [63]. *Pvt1* helps increase the stability of c‐Myc (Myc proto‐oncogene protein) to inhibit the expression of *Bcl2* (B‐cell lymphoma 2), thereby promoting autophagy and apoptosis [63]. The research showed that the downregulation of *Pvt1* significantly improved mitochondrial quantity and muscle fibre cross‐sectional area [63].

LncRNA *RP11‐253E3.3* was found to be highly expressed in muscles of spinal cord injury (SCI) patients, and it could promote *FoxO3* expression by absorbing *miR‐1207‐5p*, suggesting the role of *RP11‐253E3.3* in promoting muscle atrophy after SCI [64].

### 4.3 LncRNA in Ageing‐Induced Muscle Atrophy

Skeletal muscle weight, cross‐sectional muscle fibre area, muscle strength and muscle metabolic capacity all decline significantly with ageing, a condition known as sarcopenia. Zheng et al. found that *PRKG1‐AS1* was highly expressed in elderly muscles [65]. Interference with *PRKG1‐AS1* could promote the viability of human skeletal muscle myoblasts and reduce cell apoptosis [65]. At the same time, interference with *PRKG1‐AS1* restored the expression of myogenic differentiation antigen (*MyoD*), myogenin, *MEF2C* and *Myf5* (myogenic factor 5) [65]. *lncDLEU2* is also a potential marker for ageing‐related sarcopenia. It was upregulated in sarcopenia models and served as a ‘sponge’ for *miR‐181a* to promote *SEPP1* (selenoprotein P) expression, thereby inhibiting muscle differentiation and regeneration [66].

Our previous research found a lncRNA highly expressed in skeletal muscle named *SYISL*, which is transcribed from the fourth intron of synaptopodin‐2 gene [48]. We found that *SYISL* inhibits the expression of *p21* and myogenin by binding to PRC2 protein, thereby inhibiting muscle growth and regeneration [48]. In addition, we found that *SYISL* was upregulated in muscle atrophy models, including sarcopenia, fasting‐induced muscle atrophy and Dex‐induced muscle atrophy. Our further study showed that *SYISL* promotes muscle atrophy by acting as a molecular sponge of *miR‐103‐3p*, *miR‐205‐5p* and *miR‐23a‐3p*, thereby alleviating the inhibition of target genes like *MuRF1*, *FoxO3a* and *Atrogin‐1* [69]. We also found that *SYISL* is highly conserved across species and that human *SYISL* promotes muscle atrophy in human skeletal muscle cells and aged mice [67]. Our study provides a potential target for ageing‐induced muscle atrophy treatment.

LncRNA *Chronos* has been proven to be associated with ageing‐induced muscle atrophy, and its expression increases with ageing [68]. *Chronos* was significantly downregulated by the activation of AKT1 [68]. *Chronos* recruits EZH2 (enhancer of zeste homologue 2) to the *BMP7* locus to inhibit *BMP7* expression. [68]. Knockdown of *Chronos* can significantly increase *BMP7* expression, increase the protein synthesis rate and reduce protein ubiquitination [68]. In addition, overexpression of *Chronos* can indeed be observed to have a phenotype of impaired myogenesis, suggesting a positive regulatory role of *Chronos* in ageing‐induced muscle atrophy [68].

The expression of lncRNA *MAR1* is greatly reduced in elderly mice [69]. Wnt family member 5a (Wnt5a) is an important regulatory factor in the myogenic process. It was found that overexpression of *MAR1* could significantly increase the level of Wnt5a protein in myoblasts and skeletal muscles by binding with *miR‐487b*, thereby improving skeletal muscle mass in mice and alleviating muscle atrophy induced by ageing [69].

*lnc‐ORA* was significantly elevated in elderly mice, and it could promote myoblast proliferation and inhibit differentiation [70]. The mechanism study demonstrated that *lnc‐ORA* could repress the PTEN‐mediated PI3K/AKT signalling pathway by absorbing *miR‐532‐3p* to promote muscle atrophy and inhibit myogenic differentiation [70, 71]. Additionally, *lnc‐ORA* could directly bind to the RNA binding protein IGF2BP2 (insulin‐like growth factor 2 mRNA‐binding protein 2) and reduce the stability of myogenic genes, such as *MyoD* and *MyHC* (myosin heavy chain) [70].

### 4.4 LncRNA in Weightlessness‐Induced Muscle Atrophy

The underlying cause of muscle atrophy because of weightlessness is similar to that caused by disuse and long‐term physical inactivity, primarily involving reduced neural activity and mechanical stress. Hindlimb suspension (HLS) is a method of simulating weightlessness. In HLS mice, *lncMUMA* is significantly downregulated in muscles [72]. The expression pattern of *lncMUMA* in myoblasts cultured in a microgravity simulation environment is consistent with that in HLS mice. Notably, lncMUMA is downregulated over time in this environment and stabilizes at a certain stage [72]. Research on the regulatory mechanism indicated that *lncMUMA* could promote the expression of the *MyoD* gene by binding to *miR‐762* [72]. Skeletal muscle‐specific overexpression of *lncMUMA* can significantly increase the expression of *MyoD* in the gastrocnemius of muscle‐specific *miR‐762* knock‐in mice and effectively reverse muscle atrophy after weightlessness [72].

### 4.5 LncRNA in Cachexia‐Induced Muscle Atrophy

Cachexia is a frequent consequence of several chronic diseases and cancer, often accompanied by symptoms of muscle atrophy. Chronic kidney disease (CKD) is characterized by abnormal renal structure and function and is associated with cachexia‐induced muscle atrophy. LncRNA *Atrolnc‐1* is highly expressed in the muscles of CKD patients. *Atrolnc‐1* could promote *MuRF‐1* expression by binding to NF‐κB inhibitory factor ABIN‐1 [73]. *Atrolnc‐1* knockout can effectively alleviate the phenotype of muscle atrophy in CKD mice models [73]. In addition, *H19*, *HOTAIR*, *MALAT1* and *PVT1* are highly expressed in the kidneys of CKD patients and are associated with muscle atrophy but do not directly mediate the pathogenesis of muscle atrophy in CKD patients [90]. Patients with chronic obstructive pulmonary disease often experience severe muscle atrophy and muscle dysfunction. Lewis et al. found that *H19* was upregulated in the quadriceps of patients with COPD (chronic obstructive pulmonary disease), and its expression was inversely associated with the mass and strength of the quadriceps [91].

Recent studies have demonstrated that lncRNA *HOTAIR* was elevated in bladder tumours following cisplatin treatment [74]. That is because cisplatin treatment enhances the transcriptional activity of NF‐κB via the epidermal growth factor receptor (EGFR)/progenitor T (ProT)/NF‐κB/*HOTAIR* axis to promote *HOTAIR* expression [74]. Furthermore, *HOTAIR* facilitates IκBα degradation and enhances NF‐κB activation, thereby creating a feed‐forward regulatory circuit between NF‐κB and *HOTAIR*. *HOTAIR* and NF‐κB jointly promote the expression of inflammatory factors, which ultimately leads to muscle atrophy [74]. The study suggests the therapeutic potential of *HOTAIR* in muscle atrophy caused by bladder cancer. *MALAT1* and *lincRNA‐p21* may also play roles in muscle atrophy correlated with cancer cachexia [92].

Glucose and energy metabolism disorders caused by obesity and diabetes are also important reasons for muscle atrophy, and numerous lncRNAs mediate this process. Zhang et al. identified differentially expressed lncRNAs in skeletal muscle of *db/db* mice and normal mice [93]. Pathway analysis and gene set enrichment analysis revealed that these lncRNAs were involved in skeletal muscle function and fatty acid metabolism signalling pathway [93]. They also characterized two candidate lncRNAs, *Gm15441* and *3110045C21Rik*, that may play a dominant role in insulin resistance and its induced muscle atrophy [93]. Yu et al. identified three candidate lncRNAs for diabetes‐associated muscle atrophy [94]. Among them, *Gm20743* was correlated with the regulation of mitochondrial function and redox homeostasis in skeletal muscle [94].

Currently, research on lncRNAs directly mediating muscle atrophy in cachexia is limited. However, nearly all lncRNAs that play a positive regulatory role in the process of muscle atrophy have the potential to participate in the pathways leading to muscle mass loss in cachexia. There are indeed a few reports on lncRNAs that play roles in cachexia, and their specific roles in the progression of muscle atrophy require further investigation.

### 4.6 LncRNA in Myogenesis

*LncEDCH1* was identified in fast‐growth‐rate broiler chicken and has been shown to promote myoblast proliferation and inhibit myogenic differentiation [75]. *LncEDCH1* knockdown in gastrocnemius significantly reduced muscle mass and mean cross‐sectional area [75]. Mechanism studies showed that *LncEDCH1* enhances the activity and stability of SERCA2 (sarcoendoplasmic reticulum Ca^2+^ ATPase‐2) protein by binding to it and further achieves Ca^2+^ homeostasis to improve mitochondrial efficiency, thereby improving muscle atrophy [75]. In addition, *LncEDCH1* can also inhibit mTOR signalling to activate autophagy [75]. The increase in basal autophagic flux has been reported to improve muscle atrophy [95]. LncRNA *ZFP36L2‐AS* was found to be highly expressed in fast‐growth‐rate broiler chickens, with the ability to inhibit myoblast proliferation and promote myoblast differentiation [76]. *ZFP36L2‐AS* knockdown facilitated Ser2488 phosphorylation of mTOR, thereby inactivating the ubiquitin‐proteasome system and the autophagy‐lysosomal system [76]. Furthermore, *ZFP36L2‐AS* can also induce PC (pyruvate carboxylase) ubiquitination degradation by enhancing ACACA (acetyl‐CoA carboxylase alpha) activity, thus activating the fast‐twitch muscle phenotype and inducing muscle atrophy [76]. *LncIRS1* is significantly upregulated in fast‐growth‐rate broiler chickens, and it promotes myoblast proliferation and differentiation [77]. *LncIRS1* promotes the expression of insulin receptor substrate 1 protein by binding to miR‐15 and thus activates the IGF‐1/PI3K/AKT signalling [77]. *LncIRS1* overexpression can reduce the expression of *Atrogin‐1* and *MuRF1* and increase muscle mass and mean cross‐sectional area in 1‐day‐old chicks [77]. LncRNA *SMUL* promotes myoblast proliferation and inhibits myogenic differentiation [78]. *SMUL* overexpression upregulated the expression levels of *Atrogin‐1* and *MuRF1* and activated autophagy, whereas *SMUL* knockdown promoted muscle fibre hypertrophy [78]. Although *SMUL* has the ability to encode small peptides, its impact on myogenesis is not mediated through its protein products [78]. Mechanism studies showed that *SMUL* sORF (short open reading frame) region inhibits the mRNA expression of SMURF2 (SMAD‐specific E3 ubiquitin ligase 2) through the NMD (nonsense‐mediated decay) mechanism and thereby activates SMAD2/3 [78]. *Lnc‐mg* can promote the differentiation of MuSCs (muscle stem cells) [79]. In vivo, *lnc‐mg* skeletal muscle‐specific transgenic mice showed higher muscle mass and better athletic performance and could alleviate partial muscle mass loss after denervation induction [79]. *Lnc‐mg* contains *miR‐125b* binding sites, which have been reported to inhibit myogenic differentiation by directly targeting *IGF‐2* [79]. Another target of lnc‐mg is *miR‐351‐5p*, which inhibits lactamase β expression [80]. Knockdown of *miR‐351‐5p* promotes muscle hypertrophy in the gastrocnemius muscles of mice [80]. LncRNA *MYH1G‐AS* was found to be highly expressed in fast‐twitch fibres and was associated with open chromatin [11]. *MYH1G‐AS* promotes myoblast proliferation and inhibits myogenic differentiation. *MYH1G‐AS* overexpression reduced gastrocnemius mass and increased the expression of F‐Box Protein 25 (FBXO25), which is an ubiquitin E3 ligase involved in the UPS [11]. *MYH1G‐AS* binds to fibroblast growth factor 18 (FGF18) to inhibit FGF18 protein stability and reduce the interaction between FGF18 and SMARCA5 (SWI/SNF related, matrix associated, actin dependent regulator of chromatin, subfamily a, member 5), thereby inhibiting the binding of POU2F1 (POU domain class 2 transcription factor 1) to *SMAD4* promoter [11].

The above‐mentioned lncRNAs all regulate the process of muscle regeneration, and their abnormal expression or reduction can lead to insufficient muscle fibre regeneration, thereby causing muscle atrophy. Interestingly, some of these lncRNAs are also associated with the transformation between fast‐twitch and slow‐twitch myofibres. Fast‐twitch and slow‐twitch myofibres have different metabolic characteristics, and fast‐twitch myofibres may be more prone to muscle atrophy [76].

## 5 Therapeutic Potentials of LncRNA in Muscle Atrophy and Dystrophy

### 5.1 Finding Functional LncRNA in Muscle Atrophy and Dystrophy

A large number of lncRNAs have been found to play crucial roles in a wide range of diseases including muscle atrophy. A prerequisite issue is how to find the functional lncRNAs that are closely tied to the muscle disease in complex pathogenesis (Table 2). LncRNAs have more spatiotemporal specificity than protein‐coding genes, making them more likely to generate more alternative splicing events to regulate complex biological processes. Therefore, identifying alternative splicing events and different types of transcripts of lncRNAs during muscle atrophy is noteworthy for deeply understanding the pathogenesis of myopathy and muscle atrophy. The application of long‐read sequencing technology may become a favourable approach for exploring functional lncRNA splice variants [96]. Single‐cell RNA sequencing helps explore overlooked key lncRNAs in cell subpopulations [97]. In addition, Huang et al. developed a new algorithm called lncHOME that can identify functionally conserved lncRNAs in different species [98]. In the future, exploring the biological mechanisms of lncRNAs can benefit from the use of more advanced technologies. ARTR‐seq and RT&Tag have the characteristics of low sample demand, and they can be utilized for the analysis of single‐cell lncRNA‐protein interactions and lncRNA–chromatin interactions [99, 100]. Moreover, the epigenetic level of lncRNA itself is also worth paying attention to. Studies showed that m6A modifications could induce the formation and stability of lncRNA *Olfr29‐ps1* [105]. msRRBS can be utilized to analyse methylation heterogeneity in cell subpopulations [101]. The function of lncRNAs with low expression levels is often overlooked. These lncRNAs play a significant role far beyond the limitation of their expression level. They form membraneless compartments, namely lncRNA condensates, through phase separation mechanisms by multivalent binding to proteins and promoting multivalent interactions with other proteins, thereby regulating cellular function [106]. For instance, lncRNA *NORAD* multivalently binds to Pumilio (PUM) proteins, resulting in a higher concentration of PUM in the area occupied by *NORAD* than in the surrounding areas. This binding promotes PUM‐PUM interactions to form phase separation compartments, effectively inhibiting PUM activity and preventing genomic instability [107]. Recent studies have found dysregulated behaviour of biomolecular condensates in cancer, neurodegeneration and cardiomyopathy [108]. Therefore, it is also essential to identify the dysregulated condensates associated with muscle atrophy and their key lncRNAs. By using proximity labelling techniques, including APEX and BioID, as well as hybridization‐proximity labelling techniques, the components of dynamic or relatively stable condensates can be labelled, and further enrichment and sequencing can reveal key lncRNAs in the condensates [102]. In addition, changes in the low expression levels of lncRNAs are difficult to quantify by using traditional methods. Super resolution imaging techniques such as structural illumination microscopy can directly quantify the abundance of lncRNA in condensates at the nanometre level [103]. Droplet digital polymerase chain reaction is beneficial for accurately determining the copy number of low‐abundance lncRNAs [104].

**TABLE 2:** Techniques for finding functional lncRNAs in muscle atrophy and dystrophy.

Technology	Function	Reference
Long‐read sequencing technology	Explore functional lncRNA splice variants.	[96]
Single‐cell RNA sequencing	Explore overlooked key lncRNAs in cell subpopulations.	[97]
lncHOME algorithm	Identify functionally conserved lncRNAs in different species.	[98]
ARTR‐seq and RT&Tag	Analyse single‐cell lncRNA‐protein interactions and lncRNA‐chromatin interactions.	[99, 100]
msRRBS	Analyse methylation differences of lncRNA in cell subpopulations.	[101]
APEX and BioID; hybridization‐proximity labelling techniques	Label components of dynamic or relatively stable condensates and reveal key lncRNAs in condensates through enrichment and sequencing.	[102]
Structural illumination microscopy	Directly quantify the abundance of lncRNA in condensates at the nanometre level.	[103]
Droplet digital polymerase chain reaction	Accurately determine the copy number of low‐abundance lncRNAs.	[104]

### 5.2 Clinical Applications Potential of LncRNA in Muscle Atrophy and Dystrophy

Compared with protein‐coding genes, lncRNA has higher tissue specificity and disease specificity and can serve as specific biomarkers or therapeutic targets for specific organs or diseases. The expression changes of lncRNA often occur earlier than those of the coding genes and can be detected in the early stages of the disease, which is more conducive to early diagnosis and intervention treatment of the disease. The structure of lncRNA is simple and modular, consisting of separable functional ‘elements’ that interact with DNA, mRNA, miRNA and proteins at multiple regulatory levels [109]. Through structural or functional analysis, more targeted therapeutic drugs can be designed. In addition, lncRNA, as a nucleic acid molecule, usually has low immunogenicity. RNA therapy aims to treat diseases by targeting or utilizing RNA molecules. lncRNA has broad prospects in RNA therapy because of its advantages.

The treatment of lncRNA is mainly achieved by delivering therapeutic lncRNA into the body or removing pathogenic lncRNA. Lentiviral vectors and adenoviral vectors are commonly used lncRNA‐carrying delivery systems, and adenovirus vectors have been used in the clinic [110]. Lipid nanoparticles, the next generation of nanoparticles and extracellular vesicles are more gentle and suitable for in vivo delivery of lncRNA [104]. Finding new delivery systems that are specific, stable and gentle remains a top priority in the coming years. At present, some therapies based on antisense oligonucleotide (ASOs) have been widely applied in the clinical treatment of muscle diseases, such as Nusinersen, which alters the splicing of SMN2 gene and increases the production of full‐length SMN protein to treat spinal muscular atrophy [111]. Similarly, Eteplirsen is an ASO modified from PMO (phosphorodiamidate morpholino oligomers) that binds to the exon 51 of the pre‐mRNA of muscular dystrophy protein, thereby producing internally truncated muscular dystrophy protein [112]. LNA (locked nucleic acid) gapmers and peptide nucleic acids (PNAs) represent third‐generation ASO technologies frequently used to target lncRNAs in mice. Amodio et al. found that gymnotic delivery of MALAT1‐targeting 16mer LNA gapmeR g#5 exhibits significant antitumour activity in a humanized murine model of multiple myeloma [113]. These technologies can be applied in muscle therapy targeting lncRNA in the future. d'Ydewalle et al. found that targeted degradation of *SMN‐AS1* with ASO increases SMN expression in the mouse central nervous system [58]. Woo et al. designed an ASO called RN‐0005, which inhibits the inhibitory effect of the PRC2: SMN‐AS1 complex on SMN transcription by binding to *SMN‐AS1* [114]. In addition, using the inspiration of peptide‐facilitated macromolecular delivery to design AGR‐H19 (H19 RNA oligonucleotides conjugated with Agrin) for drug delivery does increase the level of dystrophin protein in *Dmd* C3333Y mice and alleviate some symptoms [87]. There are no mature drugs based on siRNA targeting lncRNAs for treating muscle atrophy currently, perhaps because of off‐target effects. Small molecules can serve as drugs targeting lncRNA to avoid off‐target effects. Aguilar et al. designed a small molecule drug X1 that can engage the RepA motif of lncRNA *Xist* to disrupt its conformation and thus blocks the initiation of X‐chromosome inactivation [115]. Ribonuclease targeting chimeras (RiboTACs) are bifunctional molecules that can simultaneously bind to lncRNA and recruit ribonucleases to degrade specific lncRNAs, enabling the selective degradation of pathogenic lncRNAs [116].

## 6 Conclusions and Future Perspectives

In conclusion, lncRNAs are closely correlated with muscular dystrophy and muscle atrophy induced by various factors. The complexity of the lncRNA regulatory mechanisms determines that there is still a lot of research space for them in muscle atrophy and dystrophy. Although no mature lncRNA‐based drugs have yet been developed, their unique functions in organisms continue to attract attention. We should maintain a positive attitude towards this, aiming to combine precise identification of lncRNAs with emerging therapy technologies, in order to achieve resistance to muscular dystrophy and muscle atrophy induced by various types of pathogenesis.

## Conflicts of Interest

The authors declare no conflicts of interest.
